# Cognitive Performance and Heart Rate Variability: The Influence of Fitness Level

**DOI:** 10.1371/journal.pone.0056935

**Published:** 2013-02-20

**Authors:** Antonio Luque-Casado, Mikel Zabala, Esther Morales, Manuel Mateo-March, Daniel Sanabria

**Affiliations:** 1 Departamento de Psicología Experimental, Universidad de Granada, Granada, Spain; 2 Departamento de Educación Física y Deportiva, Universidad de Granada, Granada, Spain; 3 Universidad Miguel Hernández, Elche, Spain; Université de Montréal, Canada

## Abstract

In the present study, we investigated the relation between cognitive performance and heart rate variability as a function of fitness level. We measured the effect of three cognitive tasks (the psychomotor vigilance task, a temporal orienting task, and a duration discrimination task) on the heart rate variability of two groups of participants: a high-fit group and a low-fit group. Two major novel findings emerged from this study. First, the lowest values of heart rate variability were found during performance of the duration discrimination task, compared to the other two tasks. Second, the results showed a decrement in heart rate variability as a function of the time on task, although only in the low-fit group. Moreover, the high-fit group showed overall faster reaction times than the low-fit group in the psychomotor vigilance task, while there were not significant differences in performance between the two groups of participants in the other two cognitive tasks. In sum, our results highlighted the influence of cognitive processing on heart rate variability. Importantly, both behavioral and physiological results suggested that the main benefit obtained as a result of fitness level appeared to be associated with processes involving sustained attention.

## Introduction

Recent years have shown a growing interest in the study of the relation between cognitive performance and heart rate variability (HRV). In the majority of these studies, cognitive performance is assessed by means of computer-based tasks that require participants to give fast and/or accurate responses [1]. HRV is a simple and noninvasive measurement of interactions between the autonomic nervous system (ANS) and the cardiovascular system. The analysis of the HRV is based on the study of temporal oscillations between heartbeats. The time series of HRV are obtained from the electrocardiogram, identifying the occurrence of each R wave (belonging to the QRS complex) and calculating the elapsed time between two consecutive R waves. The HRV analysis consists of a series of measurements of successive RR interval variations of sinus origin which provide indirect information about the autonomic tone [2], [3]. Thus, HRV has been used as an index of the regulation of the cardiovascular system by the ANS [2], [4], [5]. Investigating how HRV changes as a function of the cognitive task at hand provides important insights regarding the relation between cognitive and physiological processes. Here, we aimed at providing novel evidence of that relation measuring the effect of three cognitive tasks tackling different cognitive processes on the HRV of two groups of participants with different level of physical fitness.

Cognitive processing has been shown to influence HRV. For instance, Mukherjee et al. [6] showed that different levels of mental workload had differential effects on HRV (i.e., the greater the cognitive load the lower the HRV) [7]–[10]. Relevant here is the study by Luft et al. [11] who compared participants' HRV on a range of computerized cognitive tasks (the CogState cognitive battery) that involved different cognitive processes. Their results indicated significant differences in HRV between executive and non-executive tasks (executive tasks are those involving executive control that refers to the cognitive mechanism responsible for action planning, developing expectancies, automatic response inhibition and error detection [12], [13]). Specifically, the executive tasks elicited lower values of HRV compared to other tasks. Note, however, that the CogState cognitive battery consists of five tasks (simple reaction time, choice reaction time, working memory, short-term memory and sustained attention), each one presented for a very short period of time and consisting of very few trials. This can be considered a limitation in this study, because the evaluation of certain cognitive processes typically requires longer time intervals (e.g., the sustained attention task lasts only 90 seconds in the CogState). In any case, it would appear from the above that participant's HRV seems to be a suitable index of the relation between cognitive and physiological processes.

While recent research supports the sensitivity of HRV to cognitive processing, the role of physical fitness level in that relation remains unknown. However, participants' physical fitness level has been shown to influence their cognitive performance and their HRV. In effect, regular physical activity (which results in an increased physical fitness level) produces an enhanced vagal tone, which may contribute in part to the lower resting heart rate and, consequently, to higher values of HRV as a result of physiological adaptations induced by training [14]. On the other hand, regular exercise has been shown to elicit beneficial changes in brain structures and consequently, in cognitive performance [15]–[19].

Two main aims motivated the present research. First, to replicate previous studies showing the influence of cognitive performance on participants' HRV. Second, to investigate the role that participants' fitness level may play on the influence of cognitive performance on their HRV. To accomplish our goals, we compared a group of participants with a high level of physical fitness with a group of participants with sedentary lifestyle. Both groups had to perform three cognitive tasks (at rest): the psychomotor vigilance task, a temporal orienting task, and a duration discrimination task (see Methods for details).

The cognitive tasks used in the present study were selected on the basis of two main aspects. On one hand, all tasks fell within the time domain. Some of the brain structures that appear to be related to temporal and motor processing are the cerebellum and the basal ganglia [20], which are clearly involved in tasks that require an accurate representation of temporal information [21]. Additionally, aerobic training has been shown to modulate the functioning of these brain areas [19], [22], [23]. On the other hand, the few studies relating the effect of physical training on HRV and cognitive performance found that the increased in participants' HRV (as a result of training) was associated to better cognitive performance only in executive tasks [24], [25]. However, several studies support that physical exercise produces effects on performance in both executive [26] and non-executive tasks [27], [28]. Therefore, we considered important to compare participants' performance in executive and non-executive tasks. Thus, although the three tasks were framed within the time domain, each of them tackled a specific aspect of cognitive processing (i.e., sustained attention, endogenous temporal orienting of attention, and temporal resolution of visual perception).

In line with previous research [24], [25], we expected the high-fit group to have greater HRV values than the low-fit group, which would be related also with higher performance in the executive task (i.e., the temporal orienting task). Further, based on the study by Luft et al. [11], the executive task would cause the greatest reduction in the values of HRV compared to the other two tasks. Finally, we predicted that the effect on participants' HRV induced by cognitive processing would be of a larger magnitude in the low-fit group compared with the high-fit group since, as noted above, a high fitness level has positive effects on both cognitive performance and HRV.

## Methods and Design

### Ethics Statement

This study was approved by the ethics committee on human research of the University of Granada, Spain (No. 689) and complied with the ethical standards laid down in the 1964 Declaration of Helsinki. Before the start of the experimental session the participants read and signed an informed consent statement. Only in one case the participant was minor (17 years and 11 months old at the moment of collecting the data). Following the ethical standards of the local committee, the minor's parents signed a written informed consent. They were informed about their right to leave the experiment at any time. Each participant received detailed information regarding the purpose of the study at the end of the experimental session. All participants' data were analyzed and reported anonymously.

### Participants

We recruited 28 young males to participate in the present study, 14 undergraduate students from the University of Granada, Spain (all males; age range: 17–23 years old; mean age: 19.5 years old) with a low level of physical fitness (according to normative values proposed by the American College of Sports Medicine [29]), and 14 young adults with a high level of physical fitness (all males; age range: 18–29 years old; mean age: 20.7 years old), 11 from the under-23 Andalucía Cycling Team and 3 from the Faculty of Physical Activity and Sport Sciences (University of Granada, Spain; see Table 1). Two of the participants, (one from each group) were excluded from subsequent data analyses after the incremental physical test. A VO_2max_ of 46.7 ml•kg^−1^•min^−1^ was obtained for the participant from the low-fit group, a value that was not high enough to include this participant in the high-fit group but high enough to be considered as an outlier in the group of low-fit participants (mean VO_2max_ = 36.19±5.5 for the remaining 13 low-fit participants). The other participant had a VO_2max_ of 48.5 ml•kg^−1^•min^−1^, rather lower than expected for a participant in the group of high-fit participants (mean VO_2max_ = 69.05±5.6 for the remaining 13 high-fit participants). The results including the 28 participants did not differ significantly from those reported in this manuscript. However, we decided to exclude these two participants to maintain the homogeneity of the groups in terms of physical fitness level. All participants had normal or corrected to normal vision.

**Table 1:** Anthropometrical and physiological characteristics of the 26 participants included in this study.

Variables	Mean ± standard deviation	
	High-fit group	Low-fit group
***Anthropometrical characteristics***		
Sample size	13	13
Height (cm)	176.31±4.7	176.77±5.8
Weight (kg)	66.02±5.3	72.41±12.6
Body fat (%)	9.24±3.1	15.01±9.8
***Baseline parameters***		
RRi baseline (ms)	1153.7±200.8	925.69±119.3
HR baseline (bpm)	54.67±10.5	66.68±8.5
***Incremental test parameters***		
Average cadence (rpm)	90.68±8.5	69.75±7.7
Power max (W)	371.54±41.6	189.23±33.3
Relative power (W/kg)	5.63±0.5	2.65±0.5
HR max (bpm)	193.1±4.9	183.62±10.29
Blood lactate baseline (mmol/l)	1.24±0.3	1.15±0.3
Blood lactate max (mmol/l)	9.75±2.9	9.33±1.7
VO_2max_ (ml/kg/min)^a^	69.05±5.6	36.19±5.5
Normative values for VO_2max_ b	Percentile 90	Percentile 25

a VO_2max_ (ml•kg^−1^•min^−1^) = 1.8 (work rate)/(BM)+Resting VO_2_ (3.5 ml•kg^−1^•min^−1^)+Unloaded cycling (3.5 ml•kg^−1^•min^−1^). Work rate = kg•m•min^−1^ and BM = body mass (kg) [29].

b Percentile values for maximal oxygen uptake (ml•kg^−1^•min^−1^) in men. Percentile rankings: well above average (90), above average (70), average (50), below average (30) and well below average (10). VO_2max_ below 20^th^ percentile for age and sex is indicative of a sedentary lifestyle [29].

### Apparatus and materials

Participants were fitted with a FirstBeat Bodyguard monitor (Firstbeat Technologies, Oy Jyväskylä, Finland) to record their HRV during the experimental session. To describe the participants' anthropometrical characteristics we used the In-Body 230 (Biospace, Seoul, Korea). Participant completed an incremental test to determine their fitness level accurately. We used a SRM lab ergometer (Germany) to induce physical effort and obtain power values, and a Lactate Pro Meter Set (ARKRAY, Inc., Japan) to measure blood lactate concentration (see procedure below).

We used a 15.6″ LCD HP laptop PC and the E-Prime software (Psychology Software Tools, Pittsburgh, PA, USA) to control for stimulus presentation and response collection. The centre of the laptop screen was situated at 60 cm (approx.) from the participants' head and at his eye level. The device used to collect responses was the PC keyboard.

### Procedure

The experimental protocol consisted of a single session with three different phases. HRV was recorded during the entire process. In the first phase, a brief preliminary anthropometric study of each participant was performed to measure his height, weight and body fat percentage (Table 1). Subsequently, each participant rested for ten minutes in a supine position to record the baseline HRV. Participants were encouraged to stay as relaxed as possible during this procedure. During the second phase, participants performed three cognitive tasks involving temporal processing: the psychomotor vigilance task, a temporal orienting task, and a duration discrimination task. The tasks are detailed in the following section. The order of presentation of the tasks was counterbalanced across participants. Verbal and written instructions were given to the participant prior to the start of each task. The timestamp of the start and end of each cognitive task was taken for further analysis of HRV. During this phase, the participant was seated in front of the computer. Both the baseline HRV and performance in the cognitive tasks were measured in a dimmly iluminated room, at a comfortable temperature, and isolated from external noise.

Finally, in the third phase, all participants performed an incremental cycle-ergometer test to evaluate their fitness level. In order to avoid the influence of physical effort on cognitive performance [30], the incremental test was performed in the final part of the experimental session. First, the participants were exposed to a 5 min warm-up with 100 W of load. The graded maximal exercise test started at 120 W and was followed by an incremental protocol with the work rate increasing at a rate of 30 W every 2 minutes until maximal exhaustion. Each participant set their preferred cadence during the warm-up. They were asked to maintain this cadence throughout the protocol. The ergometer software was programmed to increase the load automatically. The pedal rate, load, heart rate and time of the test were continuously recorded and participants were verbally encouraged to achieve their maximal level (all participants reached the exhaustion peak). The blood lactate concentration was measured at baseline (before starting the test) and 3 minutes after stopping the test to determine the maximum concentration. Blood samples were taken from the earlobe.

The fitness level of the participants was determined from the data set obtained during the incremental physical test (see Table 1).

#### Experimental tasks

Psychomotor vigilance task: The procedure of this task was based on the original created by Wilkinson and Houghton [31]. This task was designed to measure sustained (vigilant) attention by recording participants' reaction time to visual stimuli that occur at random inter-stimulus intervals [31]–[33]. In each trial, a red circumference (6.68°×7.82°) appeared on the screen in a black background. Later, in a random time interval (from 2000 to 10000 ms), the circumference began to be filled in a red colour and in a counter-clockwise direction with an angular velocity of 0.094 degrees per second. The participants were instructed to respond as fast as they could to stop it. They must respond with their dominant hand by pressing the space bar on the PC. Feedback of the response time was displayed on the screen on each trial. The next trial began after 1500 ms. Response anticipations were considered as errors. Participants were allowed 3750 ms to respond. If a response was not made during this time, the message “You did not answer” appeared on the screen. The task comprised a single block of 10 minutes.

Temporal orienting task: This task was an executive task that measured the participants' ability to build-up expectancies about the moment when a particular event would occur, i.e., it measured the ability to selectively attend to a particular point in time [34], [35]. The stimuli presented in each trial were the following (all in the centre of the screen): a fixation point, a temporal cue and a target. The fixation point was a gray square (0.33°×0.33°). The temporal cue was a short red line (0.33°×1.15°) or a long red line (0.33°×2.48°). The short line predicted with a high probability (.75) that the target would appear early (after 400 ms), whereas the long line predicted with a high probability (.75) that the target would appear late (after 1400 ms). The target was the letter ‘O’ (0.95°×0.95°). The answer was given by pressing the “b” key of the PC keyboard. The participants were instructed to respond as fast as they could without anticipating, and were encouraged to use the temporal cue to get ready for the time of appearance of the target. The fixation point was shown for 500 ms and the temporal cue for 50 ms. After a short or long SOA (Stimulus Onset Asynchrony) of 400 or 1400 ms (with a 50% probability of occurrence of each SOA) the target appeared for 100 ms. The SOA matched the duration indicated by the cue in most trials (75% valid trials), whereas temporal expectation was not fulfilled in the remaining trials (25% invalid trials). Finally, the screen remained blank until the participant's response, or for 1900 ms. After this sequence, the next trial began. The task consisted of one block with 12 practice trials, followed by four blocks with 24 experimental trials each (96 trials in total). During the practice block, feedback was provided to participants indicating their RT. Whenever they made a mistake, a feedback message was displayed telling them whether they had responded before the target onset or whether they did not respond before the 1900 ms deadline. Feedback was not provided during the experimental blocks. Each experimental block comprised 18 valid trials and 6 invalid trials. Each block randomized the order of presentation of valid and invalid trials and of the 400 and 1400 SOA. The total duration of the task ranged from 12 to 15 minutes (mean of 14±0.8 minutes).

Duration discrimination task: This was a psychophysical task in which participants had to make a fine discrimination between the duration of two visual stimuli [36]. The task started with the presentation of a fixation point at the centre of the screen for a random duration between 500–1000 msec. The fixation point was a gray square (0.33°×0.33°) that remained on and steady for the whole trial. Then, two consecutive visual stimuli were presented (the sample and the comparison stimuli) with a random time interval of 500–1000 msec between them. The sample stimulus was a red “@” and the comparison stimulus a white “@” (2.20×2.58, both stimuli). There were two types of samples: a short sample (350 ms) and a long sample (1350 ms). The duration of the sample was manipulated between blocks of trials. The duration of the comparison stimulus was manipulated using the method of constant stimuli and the resulting functions were used to compute the just noticeable difference (JND, in milliseconds). The JND provided a suitable index of the temporal resolution of perception (i.e., small JNDs indicated high temporal resolution [36]). In blocks where the sample lasted for 350 ms the comparison stimulus could last for 175, 263, 298, 333, 368, 403, 438 or 525 ms. In blocks were the long sample was presented the comparison stimulus could last for 675, 1013, 1148, 1283, 1418, 1553, 1688 or 2025 ms. Participants had 5000 ms to respond before the start of the next trial.

Participants were instructed to discriminate whether the duration of the comparison stimulus was shorter or longer than the duration of the sample stimulus. If the duration of the comparison stimulus was longer than the duration of the sample stimulus, the participant should respond by pressing the up arrow. Otherwise, the participants should press the down arrow. The participants completed two ‘short-sample’ blocks and two ‘long-sample’ blocks of 32 trials each, presented in counterbalanced order. Also, within each block, trials of varying duration were counterbalanced and randomly intermixed across trials. Each of the comparison stimuli was presented a 12.5% of the total number of trials in each block. There was not feedback after each trial. In addition, rough temporal estimation data were collected. During the task, the participant had to respond twice (at the middle of the task and at the end of the task) to the following question that appeared on the screen: “How long has it been since the task started?”. The response was done by keying the number of minutes and then the task continued. The total number of trials of this task was 128 and its overall duration ranged from 10–13 minutes (mean of 11±1 minute). In this case, accuracy was stressed over response speed.

#### HRV measures

Two electrodes were placed on the participant's chest about 2.5 cm below the right clavicle and between the two bottom-ribs on the person's left side. The data were collected from FirstBeat Bodyguard monitor with a sampling rate of 1000 Hz (1 ms). Subsequently, data were transferred to the FirstBeat Athlete Software (FirstBeat Technologies Oy-Jyväskylä) and each downloaded R-R interval file was then further analyzed by means of the Kubios HRV Analysis Software 2.0 (The Biomedical Signal and Medical Imaging Analysis Group, Department of Applied Physics, University of Kuopio, Finland) [37].

The recordings were preprocessed to exclude artifacts by eliminating RR intervals which differed more than 25% from the previous and the subsequent RR intervals [38]. Removed RR intervals were replaced by conventional spline interpolation so that the length of the data did not change (i.e., resulting in the same number of beats). We used the smoothness prior method with a Lambda value of 500 to remove disturbing low frequency baseline trend components [39].

The method of analysis of the HRV data used in this study was through linear mathematical processes (i.e., time domain method). This method is based on the mathematical calculation of the variations in time occurring between beats. The following parameters were used to analyze the HRV within the time domain: the mean R-R interval (RRi), standard deviation of R-R interval (SDNN) and the root-mean-square difference of successive normal R-R intervals (rMSSD). The denotations and definitions for the HRV parameters in this paper follow the guidelines given in *Task force of the European society of cardiology* and the *North American society of pacing and electrophysiology* [2].

### Design and data analyses

In order to match the samples in time intervals of equal duration we considered the first 10 minutes of each task allowing an accurate comparison between them (the results of the analyses with the total length of each task mimicked those presented here). This analysis also allowed the generation of three blocks of an equal duration of 200 seconds for each task (psychomotor vigilance task, temporal orienting task and duration discrimination task) and participant. One single time interval of 600 seconds was considered for the analysis of the rest baseline. In order to check for differences between the two groups regarding their fitness level, data from the different variables obtained during the incremental test were analyzed by using t-tests for independent samples.

Participants mean HRV data were transformed to their natural logarithms in order to ensure a normal distribution. The HRV, RT and accuracy data were analysed through factorial analysis of variance (ANOVA), t-test for independent samples, and the Mann-Whitney U nonparametric test when appropriate. The results of the ANOVA were further explained by t-tests for independent samples (in the case of between-subjects effects) and by pair-wise comparisons (in the case of within-participants effects). Violation of the sphericity (within-participants factors) and homoscedasticity (between-participants factor) was accounted for by applying the Greenhouse-Geisser correction (corrected p values and degrees of freedom are reported) and the Mann-Whitney U nonparametric test, respectively.

The experiment consisted of a factorial design with the between-participants variable Group (high-fit, low-fit) and the within-participants variables of Task (psychomotor vigilance task, temporal orienting task and duration discrimination task) and Block (1, 2, 3).

#### Behavioural data processing

For the psychomotor vigilance task trials with RTs below 100 ms (4.17%) were discarded from the analysis. For the temporal orienting task, only the experimental blocks were included in the analysis. In this case, we did not take into account the RTs below 100 ms and above 1000 ms (2.8%). In both cases, the first trial of the task for each participant (1.2% and 0.36%, respectively) was discarded from the analysis. For the psychomotor vigilance task, the data analyses were performed on the overall participants' mean RT, the number of lapses (i.e., errors of omission; RTs ≥500 ms [32]) and the mean of the slowest and fastest 10% RTs (i.e., average in milliseconds of the 10% of fastest and slowest trials for each participant). T-test for independent samples and an ANOVA were used to analyze the behavioural data from the psychomotor vigilance task and the temporal orienting task, respectively. The number of lapses in the psychomotor vigilance task, the rough temporal estimation and JND values in the duration discrimination task did not fit a normal distribution. The analysis of these variables was performed using Mann-Whitney U test for independent samples. The remaining variables were normally distributed according to the Kolmogorov-Smirnoff and Lilliefors tests (all *ps*>.20).

In order to compute the JND in the duration discrimination task, the data from each participant were transformed to Z scores, and the Z score distributions were fitted to linear regressions [40]. JNDs were computed for each participant using the slopes of such linear trends. Finally, the difference between the time estimated by the participants and the actual time was calculated for the analysis of the rough temporal estimation.

## Results

### Behavioural

Psychomotor vigilance task: The high-fit group responded faster overall than the low-fit group (278±22 ms and 297±21 ms, respectively), *t*(24) = 2.22, *p* = .03. The t-tests for independent samples also revealed significant differences between groups in the slowest 10% RTs, *t*(24) = 2.69, *p* = .01, (379±51 ms and 429±44 ms, for the high-fit and low-fit groups, respectively). The low-fit group was also slower in the range of the 10% fastest RTs than the high-fit group (238±17 ms and 230±11 ms for the low-fit and high-fit, respectively), although this difference failed to reach statistical significance, *t*(24) = 1.41, *p* = .17. Participants in the low-fit group committed more lapses than the high-fit group (1.1±1.2 lapses and 0.5±0.7 lapses, respectively), although again this difference did not reach significance, *U* = 61.5, *z* = −1.18, *p* = .24.

Temporal orienting task: An ANOVA with the factors of Group (high-fit and low-fit), Validity (valid, invalid), Current SOA (400, 1400) and Previous SOA (400, 1400) showed the typical results obtained with this type of tasks [41]: SOA by Validity, *F*(1,24) = 49.4, *p*<.01, *η^2^_p_* = .67, and Previous SOA by Current SOA, *F*(1,24) = 25.52, *p*<.01, *η^2^_p_* = .51. Crucially, neither the main effect of Group nor any interaction involving this factor reached statistical significance (all *ps*>.21).

Duration discrimination task: The Mann-Whitney U tests on the participants' JND data for the two sample durations did not reveal any statistical significant difference between groups (both *ps*>.18). Rough temporal estimation did not differ between groups either (both *ps*>.29).

### Physiological

The t-tests for independent samples revealed significant differences between groups in the maximum power output (watts) achieved by each participant during the incremental test, *t*(24) = 12.34, *p*<.01, and VO_2max_, *t*(24) = 15.04, *p*<.01. Both data showed evidence of the difference in fitness level between groups (see Table 1). In addition, t-tests for independent samples were also used to compare the different parameters of HRV between groups in the baseline measure. The indices RRi, *t*(24) = 3.41, *p*<.01, and rMSSD, *t*(24) = 2.10, *p*<.05 showed significant differences (see Table 2). The high-fit group showed larger SDNN values than the low-fit group, although this difference failed to reach statistical significance, *t*(24) = 1.58, *p* = .13.

**Table 2:** Mean (± standard deviation) for the HRV parameters for the two groups of participants at rest.

Parameters	Values at rest condition	
	High-fit group	Low-fit group
RRi (ms)	1153.70 (200.8)*	925.69 (119.3)*
SDNN (ms)	74.14 (25.3)	58.20 (17.9)
rMSSD (ms)	92.31 (39.3)*	61.60 (21.2)*

* p<.05 (using log-transform data).

A repeated-measures ANOVA with the between-participants factor of Group (high-fit and low-fit) and within-participants factors of Task (psychomotor vigilance task, temporal orienting task and duration discrimination task) and Block (1, 2, 3) was conducted on each HRV parameter. The ANOVA revealed a significant main effect of Group in the parameter RRi, *F*(1,24) = 8.24, *p* = .01, *η^2^_p_* = .26 (*U* = 38, *z* = 2.38, *p* = .02). However, there were not significant differences for the SDNN and rMSSD indexes (both *ps*>.12).Importantly, in all parameters the high-fit group obtained higher values than the low-fit group.

**Figure 1:**
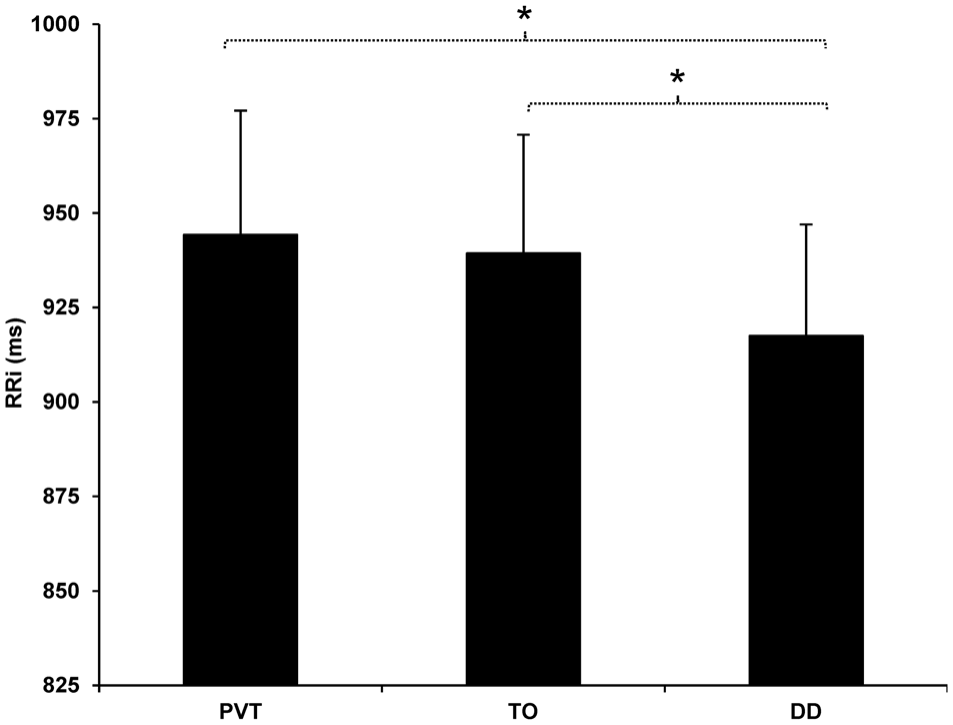
Modulation of the RRi parameter as a function of the task. Mean RR intervals in milliseconds (ms) for both groups in each of the cognitive tasks (PVT = psychomotor vigilance task; TO = temporal orienting task; DD = duration discrimination task). Bars represent standard errors of the mean. *p≤.01.

Crucially, the main effect of Task was significant for all indexes (see Table 3): RRi, *F*(2,48) = 5.66, *p*<.01, *η^2^_p_* = .19 (see Figure 1), SDNN, *F*(1.38, 33.08) = 13.72, *p*<.01, *η^2^_p_* = .36, and rMSSD, *F*(1.38, 33.04) = 4.08, *p* = .039, *η^2^_p_* = .14. Further planned comparisons revealed significant differences between the psychomotor vigilance task and the duration discrimination task in all indices: RRi, SDNN (both *ps*≤.01) and rMSSD (*p* = .036). Similarly, significant differences were found also between the temporal orienting task and the duration discrimination task in RRi (*p* = .01) and rMSSD (*p* = .038) although the difference in SDNN was not significant (*p* = .12). However, there were not significant differences between the psychomotor vigilance task and the temporal orienting task in any of the indexes (all *p*s>.17) except for the SDNN parameter (*p*<.01).

**Table 3:** Mean (± standard deviation) for the HRV indices as a function of Task.

	Psychomotor vigilance task	Temporal orienting task	Duration discrimination task
**RRi (ms)**	944.2 (190.2)^3^	939.3 (187.9)^3^	917.5 (171.6)^1^^,^ 2
**SDNN (ms)**	77.1 (28.4)^2^^,^ 3	66.8 (26.2)^1^	63.8 (24.1)^1^
**rMSSD (ms)**	71.7 (34.9)^3^	69.1 (36.2)^3^	64.2 (33.4)^1^^,^ 2

1 Significant difference with respect to the psychomotor vigilance task, p<.05.

2 Significant difference with respect to the temporal orienting task, p<.05.

3 Significant difference with respect to the duration discrimination task, p<.05.

Note: All p values correspond to log-transform data analyses.

**Figure 2:**
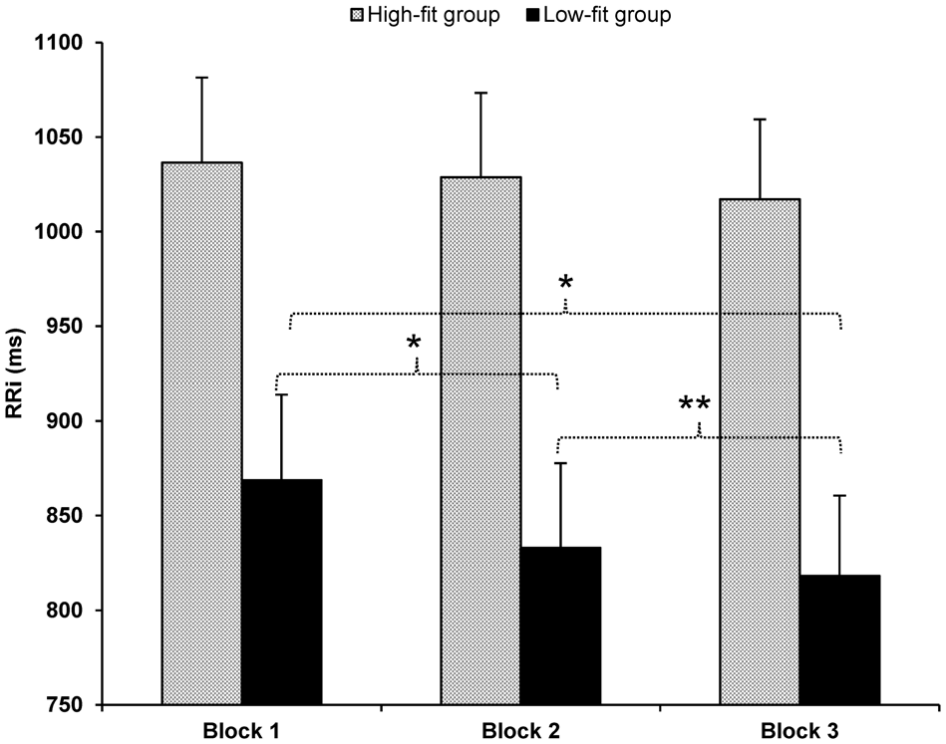
Main effect of Block for the high-fit and low-fit groups. Mean RR intervals in milliseconds (ms) for the high-fit and low-fit groups in each of the blocks of the three tasks (Block 1 = between 0 and 200 seconds of each task; Block2 = between 200 and 400 seconds of each task; Block 3 = between 400 and 600 seconds of each task). Bars represent standard errors of the mean. *p<.01; **.05<*p*<.10.

In addition, the ANOVAs revealed significant main effects of Block (all *ps*<.01, except for the SDNN, *p* = .15), that were better qualified by the significant interactions between Group and Block (see Table 4). This interaction reached statistical significance in RRi *F*(2,48) = 5.40, *p* = .01, *η^2^_p_* = .18 (see Figure 2) and rMSSD *F*(1.44, 34.61) = 5.59, *p* = .01, *η^2^_p_* = .19. In the SDNN index the interaction was marginal *F*(1.49, 35.7) = 3.49, *p* = .053, *η^2^_p_* = .13. However, in order to explain this interaction further we performed planned comparisons in all the parameters since every index followed the same common trend, i.e., the main effect of block was significant only for the low-fit group. The planned comparisons for the low-fit group showed significant differences between block 1 and block 2 in RRi and rMSSD (both *ps*≤.01) and a marginal statistical difference in SDNN (*p* = .07). When comparing block 1 with block 3 all parameters showed significant differences (all *ps*<.01). Furthermore, significant differences between block 2 and block 3 were found in rMSSD (*p* = .01) and marginal differences in RRi (*p* = .06). In this case, the difference was not significant for the SDNN index (*p* = .24). Instead, planned comparisons between blocks for the high-fit group did not reveal significant differences in any of the parameters (all *ps*>.12 except for the RRi between block 1 and 3, *p* = .07).

**Table 4:** Mean (± standard deviation) for the HRV indices as a function of Group and Block.

Parameters	High-fit group			Low-fit group		
	B1	B2	B3	B1	B2	B3
**RRi (ms)**	1036.4 (206.9)	1028.7 (206.6)	1017.0 (198.8)	868.8 (100.7)^2^^,^ 3	833.0 (96.0)^1^	818.2 (84.5)^1^
**SDNN (ms)**	78.6 (29.5)	76.9 (28.7)	79.8 (30.5)	63.6 (17.6)^3^	59.2 (21.2)	57.1 (18.8)^1^
**rMSSD (ms)**	83.8 (38.1)	84.8 (39.1)	82.3 (40.1)	59.7 (22.5)^2^^,^ 3	52.5 (25.5)^1^^,^ 3	46.8 (17.0)^1^^,^ 2

B1: first block of each task (between 0 and 200 seconds); B2: second block of each task (between 200 and 400 seconds); B3: third block of each task (between 400 and 600 seconds).

1 Significant difference with respect to B1, p<.05.

2 Significant difference with respect to B2, p<.05.

3 Significant difference with respect to B3, p<.05.

Note: All p values correspond to log-transform data analyses.

Finally, the interaction between Task and Block was also statistically significant in SDNN *F*(2.88, 69.05) = 3.26, *p* = .028, *η^2^_p_* = .12 and marginally significant in rMSSD *F*(2.59, 62.28) = 2.67, *p* = .06, *η^2^_p_* = .10. However, this interaction was not statistically significant for the RRi parameter (*p* = .28). Planned comparisons were performed in the SDNN index, where the interaction was statistically significant. These planned comparisons for the psychomotor vigilance task showed significant differences between block 1 and block 2, and also between block 1 and block 3 (both *ps*<.01). The difference between block 2 and block 3 was not statistically significant (*F*<1). Instead, planned comparisons between blocks for the temporal orienting task and duration discrimination task did not reveal significant differences (all *ps*>.09). None of the other terms in the ANOVA in any of the HRV parameters reached statistical significance (all *ps*>.13).

## General Discussion

In the present study, we investigated the relation between cognitive performance and HRV as a function of the participants' fitness level. To accomplish our goal, we measured the HRV of a group of high-fit participants and a group of low-fit participants while performing (at rest) three cognitive tasks involving sustained attention, temporal orienting of attention, and fine temporal discrimination.

The behavioural results showed better performance of the high-fit group with respect to the low-fit group in the psychomotor vigilance task (i.e., the sustained attention task [32]). These results suggest that cognitive processing involved in sustained attention was more efficient in the high-fit group than in the low-fit group. Crucially, the effect of fitness level was restricted to the sustained attention task.

The high-fit group showed greater vagal control in HRV parameters (i.e., both at rest and during performance of the cognitive tasks) presumably as a result of aerobic training [14]. Therefore, according to previous research [24], [25], one could have expected better performance of the high-fit group with respect to the low-fit group in the executive task used in our study (i.e., the temporal orienting task). However, our results did not seem to replicate those previous accounts.

It would appear then that higher values of HRV do not translate into better executive performance in all cases. Note, though, that it is possible that the level of executive demands of the temporal orienting task used here was not high enough to differentiate performance between the two groups of participants. Furthermore, the age of the participants included in this study could have also precluded a difference in performance between the high-fit and the low-fit group. Indeed, executive function may be more susceptible to improvement with physical activity in elderly populations according to previous research [24], [42]. In any case, our results seem to support the idea that aerobic training produces selective benefits in cognitive performance [43], [44]. However, future research is needed to clarify the potential role of fitness level on behavioural cognitive performance and to provide novel information to shed light into these seemingly contradictory results.

Crucially, the outcome of the present experiment showed a clear modulation of the HRV parameters as a function of the task at hand. The lowest HRV values were found in the duration discrimination task. Therefore, these results suggest that the perceptual demands of the task seem to be a key factor in the differential modulation of HRV as a function of cognitive processing. That is, it would appear that the HRV is more sensitive to perceptual demands than to (executive or sustained) attentional demands. This main effect of Task was not influenced by the level of fitness. In this regard, our results support previous studies that concluded that the association between the task demands and the autonomic modulation was independent of the baseline HRV [11].

Thayer et al., based on the extant research, have recently proposed the neurovisceral integration model to account for the links between cognitive processing and the ANS [45], [46]. This model showed a unified structural and functional network linking HRV and prefrontal neural structures, responsible of executive processing. However, to the best of our knowledge, there is not any previous study comparing the influence of performing a sustained attention task, an executive task, and a perceptual task on participants' HRV. Our results showed that the task demanding fine perceptual (temporal) discrimination was the most incisive on HRV. Therefore, our finding suggests the need to take into account the perceptual task demands as a key factor in the further development of this model. While our results seem to contradict Thayers et al.'s model (i.e., the effect of the perceptual task on HRV was larger than that of the executive task), it is important to note that previous research in Cognitive Neuroscience has revealed that prefrontal neural structures are also involved in difficult perceptual discriminations [47]. In that sense, it may be the case that the duration discrimination task used in the present study was more demanding in terms of executive control than the temporal orienting task, which would support Thayer's et al. conclusions. In any case, note that the purpose of this study was not to test the reliability of Thayer's et al neurovisceral integration model.

Another major finding of our study was the gradual decrement in participants' HRV as a function of the time spent on the task. Crucially, this influence was significant only in the low-fit group. It would appear then that decrements in sustained attention provoked by the time spent performing the cognitive tasks mainly affected the low-fit group. Taken together, both the behavioural results (i.e., better cognitive performance by the high-fit group than the low-fit group in the sustained attention task), and physiological results (i.e., the high-fit group was more resistant to the time spent on the tasks than the low-fit group, in terms of HRV decrements) suggest that the main benefit obtained as a result of fitness level appeared to be associated with processes involving sustained attention.

As noted above, the participants' HRV was also influenced by the overall time on task. All tasks had a common trend towards a gradual decrease in HRV during their time course. However, the significant interaction between Task and Block suggests that the gradual reduction of HRV as a function of the time on task depended on the type of cognitive processing involved.

The psychomotor vigilance task showed the largest reduction in HRV as a function of the time on task. This finding further supports the psychomotor vigilance task as a reliable tool to measure sustained attention. Interestingly, the reduction of HRV as a function of the time on task, and the modulation of this effect by the particular task at hand, have not been reported in previous studies. The very short duration of the cognitive tasks used in previous research, like in Luft et al.' study [11], may have prevented any decrement of HRV as a function of the time on the task.

In sum, we conclude that HRV was an excellent index of autonomic tone modulation by cognitive processing in our study, with the highest effect produced by the perceptual task. In addition, the fitness level of the participants appeared to be a key factor, with an improved functioning of the cardiac autonomic control (i.e., higher HRV values) and cognitive performance (in the sustained attention task) in the high-fit group with respect to the low-fit group. Moreover, the high-fit group appeared to be less affected by the time spent performing the cognitive tasks, which can be taken again as an index of more efficient sustained attention. Future research will determinate further the links between particular cognitive processes and HRV, and the role played by physical fitness level on this relationship.
